# Comparative Study of Growth Morphologies of Ga_2_O_3_ Nanowires on Different Substrates

**DOI:** 10.3390/nano10101920

**Published:** 2020-09-25

**Authors:** Badriyah Alhalaili, Ruxandra Vidu, Howard Mao, M. Saif Islam

**Affiliations:** 1 Nanotechnology and Advanced Materials Program, Kuwait Institute for Scientific Research, Safat 13109, Kuwait; bhalaili@kisr.edu.kw; 2 Electrical and Computer Engineering, University of California at Davis, Davis, CA 95616, USA; homa@ucdavis.edu (H.M.); sislam@ucdavis.edu (M.S.I.); 3 Faculty of Materials Science and Engineering, University of POLITEHNICA of Bucharest, 060042 Bucharest, Romania

**Keywords:** Ga_2_O_3_, nanowires, Ga oxidation

## Abstract

Gallium oxide (Ga_2_O_3_) is a new wide bandgap semiconductor with remarkable properties that offers strong potential for applications in power electronics, optoelectronics, and devices for extreme conditions. In this work, we explore the morphology of Ga_2_O_3_ nanostructures on different substrates and temperatures. We used silver catalysts to enhance the growth of Ga_2_O_3_ nanowires on substrates such as p-Si substrate doped with boron, 250 nm SiO_2_ on n-Si, 250 nm Si_3_N_4_ on p-Si, quartz, and n-Si substrates by using a thermal oxidation technique at high temperatures (~1000 °C) in the presence of liquid silver paste that served as a catalyst layer. We present the results of the morphological, structural, and elemental characterization of the Ga_2_O_3_ nanostructures. This work offers in-depth explanation of the dense, thin, and long Ga_2_O_3_ nanowire growth directly on the surfaces of various types of substrates using silver catalysts.

## 1. Introduction

Conventional silicon (Si)-based devices cannot tolerate extreme environments and cannot exhibit high thermal and chemical stability, high electrical persistence (e.g., high breakdown voltage), or high-radiation hardness. Current sensors and electronics face challenges in operating within severe environments, such as high temperature, high voltage, and power. Hence, there is a tremendous need to recognize the dynamics of such environments and to innovate the manufacturing technologies for sensing and controlling in harsh conditions.

Gallium oxide (Ga_2_O_3_) has the potential to improve a diverse range of applications in power and optoelectronics more so than other semiconductor materials such as Si, ZnO, SiC, and GaN. The material of Ga_2_O_3_ has a high-breakdown voltage and low on-resistance which has the potential to replace SiC and GaN [1]. In addition, due to the high chemical and thermal stability of Ga_2_O_3_, its exceptional properties distinguish it from other wide bandgap semiconductors. Scientists are interested in Ga_2_O_3_, among other wide bandgap materials, for several optical and sensing applications. However, several challenges related to Ga_2_O_3_ still need to be addressed. A critical issue is to grow Ga_2_O_3_-based materials at a reduced cost and simple fabrication process. A number of growth techniques have been investigated by growing Ga_2_O_3_ thin films and nanowires. These techniques involve thermal oxidation [2], pulsed laser deposition (PLD) [3,4], molecular beam epitaxy [5,6], metalorganic chemical vapor deposition [7,8] and hydrothermal synthesis [9,10,11].

Different substrates have been used to examine and produce low-cost and high-quality Ga_2_O_3_ growth and lattice mismatch between the substrate remains a big issue. Even though the Ga_2_O_3_ substrate offers lattice match, its cost is very high. Similar to the Ga_2_O_3_ substrate, the lattice constant of GaN is well matched [12]; however, high prices for GaN substrates prohibit large-scale production. Although different materials have been explored to grow Ga_2_O_3_, the lattice mismatch between the target substrate and Ga_2_O_3_ has been a major problem, seen through the sapphire (Al_2_O_3_) in-plan epitaxial relationship of β-Ga_2_O_3_ <010>||Al_2_O_3_ <1-100> and β-Ga_2_O_3_ <102>||Al_2_O_3_ <1120> with 4.2% and 10.7%, respectively [13], and MgAl_6_O_10_ with 2.9% [13,14].The second challenge is the existence of defects, causing structural and morphological problems. ε-Ga_2_O_3_ keeps its morphology and has a low-defect density when grown on GaN [12]. When using MgAl_6_O_10_, several types of point defects in the Ga_2_O_3_ have been observed, such as oxygen vacancies, interstitial Ga, Ga vacancies, and gallium-oxygen vacancy pairs. When MgAl_6_O_10_ is used as a growth substrate, the donor band is formed by pairs of gallium vacancies and gallium-oxygen vacancies [15]. When Ga_2_O_3_ is grown on MgO, it is initially amorphous and requires annealing to achieve good crystallinity [16]. However, this also decreases the bandgap, making the material less sensitive to UV light.

Although some of these semiconductors are good candidates to grow Ga_2_O_3_ films, the material, electrical and, optical properties are still not satisfactory [17,18] due to defects, structural and morphological disorders, lattice mismatch, in addition to the substrate cost. An alternative solution is required to address the current expensive growth techniques or high-cost substrates to enhance high-scale production of Ga_2_O_3_ using simple, effective, and low-cost processes. Our technique is based on performing an oxidation method to decompose GaAs wafers and to directly oxidize liquid gallium. In this study, we present the morphology of Ga_2_O_3_ nanowires grown on different substrates. The structure of nanowires and nanorods are important in electronic and optoelectronic applications such as gas sensors [19], photodiodes [20], and resistive switches [21].

## 2. Materials and Methods

Ga_2_O_3_ was grown on different substrates as follows: (100) p-Si substrate doped with boron, 250 nm SiO_2_ on n-Si, 250 nm Si_3_N_4_ on p-Si, n-Si substrate doped with phosphorus, and quartz (University wafers, South Boston, MA, USA). First, each substrate was cleaned with acetone and ethyl alcohol, rinsed with deionized water, and then dried with an N_2_ gun. To obtain Ga_2_O_3_, 0.2 g of gallium (Ga) (purity 99.999%, obtained from Sigma Aldrich, Mountain View, CA, USA) was dripped into a thin layer over a cleaned surface of substrates. To investigate the effect of Ag nanoparticles (NPs) on the growth of Ga_2_O_3_, a silver paste was used to coat the Ga layer. The results were compared with samples grown under the same conditions on bare substrates.

**Figure 1:**
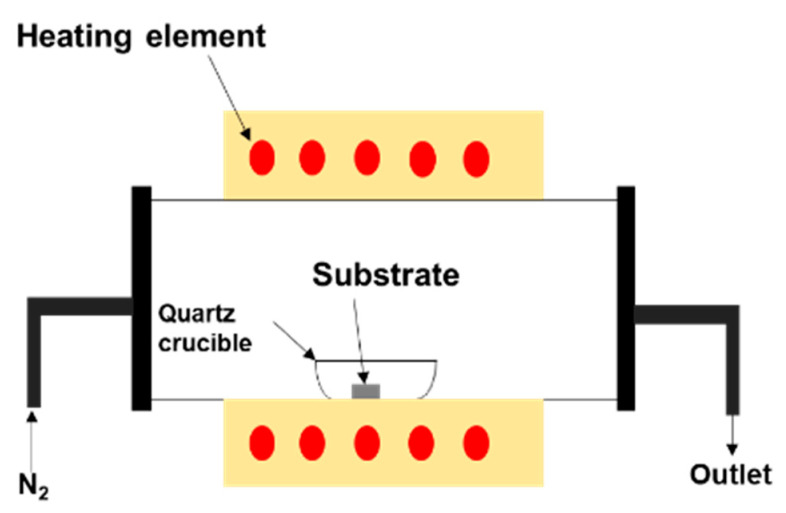
Illustration of the sample set-up inside the furnace, which was used to study the morphology of Ga_2_O_3_ on different substrates. A mixture of liquid Ga as a source and Ag as a catalyst was placed on the surface of substrates to enhance the growth of Ga_2_O_3_.

Then, the sample was loaded into a quartz crucible and placed into an OTF-1200X-50-SL horizontal alumina tube furnace made by MTI Corporation (Richmond, CA, USA). Heating occurred in a 20 sccm nitrogen atmosphere. Figure 1 illustrates the set-up of the sample inside the furnace, which was used to grow gallium oxide on different substrates.

The morphological, structural, and elemental characterization of Ga_2_O_3_ was performed using scanning electron microscopy (FEI Nova NanoSEM430, FEI Company, Hillsboro, OR, USA), while an energy dispersive X-ray spectroscopy (EDS) and high resolution transmission electron microscopy equipped with an energy-dispersive spectroscopy (EDS) profile analysis was performed to explore the growth mechanism of Ga_2_O_3_ nanostructures. A focused ion beam (FIB), equipped with an X-MaxN 50 mm^2^ Energy Dispersive X-ray Spectroscopy (EDS) from Oxford Instruments, Abingdon, UK, was used to perform EDS mapping of the surface of Ga_2_O_3_.

## 3. Results and Discussion

The growth morphologies of gallium oxide were studied on various substrates and at different oxidation temperatures. Previous work has shown that crystalline the Ga_2_O_3_ phase was obtained under the same oxidation conditions used in this work [2,22]. Because the oxidation conditions were kept constant in this work, the nanostructures formed during oxidation are primarily related to the substrate. The effect of various substrates and the processing parameters on the morphology of Ga_2_O_3_ are detailed in the following sections.

### 3.1. Growth Morphologies of Ga_2_O_3_ on Different Substrates

**Figure 2:**
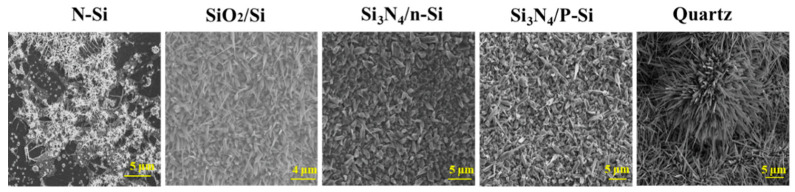
SEM images of Ga_2_O_3_ grown at 1000 °C on different substrates in the presence of the Ag catalyst. Different morphologies of Ga_2_O_3_ nanostructures were observed on the substrates used due to the effect of nanoparticle pattern formation and their coalescence, temperature, and catalyst properties.

The nature and composition of the substrate was a challenging issue in the growth of thin films due to the interface chemistry and adherence processes during oxidation. To improve the interface properties, the substrates were chemically modified to reduce the lattice mismatch, steer heterogeneous nucleation, and ensure a uniform and adherent thin film. Figure 2 presents a comparison of the morphologies of gallium oxide grown at 1000 °C on different substrates.

**Figure 3:**
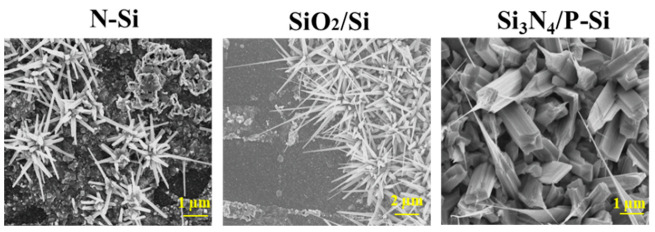
SEM images of different Ga_2_O_3_ nanostructures grown on different substrates: N-Si, SiO_2_/Si and Si_3_N_4_/P-Si.

The shape of the nanostructures was generally driven by the nuclei formed on the oxygenated gallium species [23] and Ag nanoparticles [24]. The images with low magnification provided information on the growth density on the substrate used (Figure 2), while the morphology of the nanowires could be observed at higher magnification. Figure 3 shows the different shapes of Ga_2_O_3_ nanowires observed by SEM after oxidation at 1000 °C on different Si-based substrates. Differences in nanowire morphologies were observed with these substrates. On the N-Si substrate, rod-like nanowires were observed in flower-like patches, while a high density of sharp nanowires were observed on the oxide surface. The diameter of the Ga_2_O_3_ nanowires (NWs) was about 105 ± 35 nm for both substrates, but the Ga_2_O_3_ NWs on SiO_2_/Si were at least twice the height of the nanorods grown on the N-Si substrate. We should mention that the SEM image of the Ga_2_O_3_ NWs on SiO_2_/Si was taken at the border between the Ag-coated surface and the SiO_2_ surface, which emphasizes the catalyst effect of Ag on the growth of Ga_2_O_3_ NWs [18]. Although, it has been shown that sputtered Ag thin film plays a major role in enhancing wettability of the surface and leads to a homogeneous coating of Ga_2_O_3_ nanowires [22]. Using liquid Ag on the top or bottom of liquid Ga showed a nonuniform coating of silicon or quartz as shown for N-Si and SiO_2_-Si (Figure 2 and Figure 3). The SEM image of the Ga_2_O_3_ NWs on Si_3_N_4_/P-Si presented a completely different morphology in which the nanowires were composed of thick, rectangular nanorods with a single, ultra-sharp tip on top of the rod. The shape of the nanorods was directly related to the monoclinic crystalline structure of β-Ga_2_O_3_ with their c-axis aligned in the vertical direction. Taking a closer look at the top of nanorods, it was observed that a few single crystals nanosheets stuck up to form the thick nanorods. The question as to why nanowires grow with their c-axis vertically-aligned in the absence of epitaxy was recently addressed by Azulay et al. [25], who confirmed that the direction of ZnO nanowires correlate with the electric field emanating from the substrate, i.e., the substrate surface charge affects the growth direction of polar semiconductors. Although the nanorods were much thicker than that observed on N-Si, the tip on top of the nanorods was much sharper and longer. For all Si-based substrates, the Ga_2_O_3_ NWs grew at random angles on the substrate.

The degree of supersaturation is the dominant factor that controls the morphology of growth. Nanosheets could be formed as the nuclei coalesce on the surface, forming a solid layer that stops the growth of one-dimensional nanometer-scale nuclei, favoring a thicker nanosheet-like oxide growth from liquid Ga [26]. The growth of Ga_2_O_3_ on substrates was greatly affected by the presence of an Ag thin film, which was introduced in this system to modify the local energy properties at the substrate/nucleus interface. Alhalaili et al. [22] discussed the growth mechanism of Ga_2_O_3_ nanowires in more detail. The physical interactions between Ga, Ag, and O, and the stability of various phases formed during the oxidation of Ga in the presence of Ag brings new challenges in the formulation of a unitary oxidation mechanism for the growth Ga_2_O_3_ nanostructures. Further studies are required to determine the interaction between Ga and Ag in different phases.

### 3.2. Effect of Ag Catalyst on the Growth Morphologies of Ga_2_O_3_

**Figure 4:**
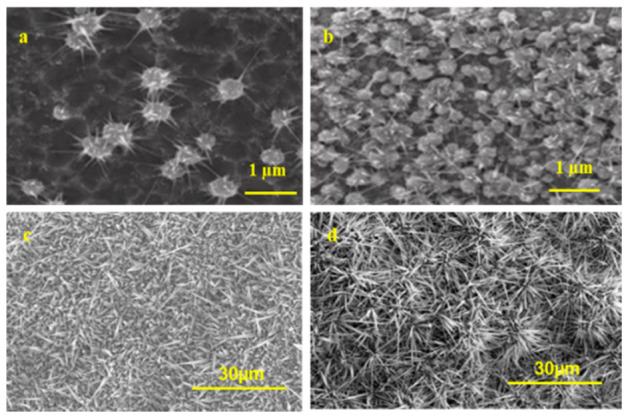
SEM images of gallium oxide on quartz, which was obtained by oxidation at different temperatures as follows: 800 °C without (**a**) and with Ag (**b**); 1000 °C without (**c**) and with Ag (**d**). The growth density of gallium oxide has significantly increased in the presence of the Ag catalyst at 800 °C. Similarly, at 1000 °C, the density and the length of Ga_2_O_3_ nanowires were greatly enhanced due to the presence of the Ag catalyst.

To investigate the growth of nanostructures on different substrates, Ga_2_O_3_ nanostructures were grown in the presence and the absence of Ag as a catalyst at 800 °C and 1000 °C. The SEM images in Figure 4 show that the density, shape, and size of nanostructures were enhanced when the oxidation of Ga occurred in the presence of Ag.

At temperatures <800 °C, Ga_2_O_3_ nucleation increased much more due to the silver catalyst presence (Figure 4a,b). Each grown particle was surrounded by tiny nanostructures in the range of 500–1000 nm, some of them similar to nanowires. However, at 1000 °C, each nanowire of these Ga_2_O_3_ particles was grown longer and denser (Figure 4c,d). The diameter of the nanowires without silver catalyst was larger than those with silver. Denser Ga_2_O_3_ nanostructures were grown at 1000 °C in the presence of an Ag catalyst compared to those grown without Ag. In contrast, the Ag-free samples had a shorter wire-like morphology with an average height of 500–5000 nm. The use of an Ag catalyst improved the length of the nanowires by an average of 10–40 µm. As a general observation, the use of Ag as a catalyst for the growth of Ga_2_O_3_ produced a high density of nanostructures (Figure S1 in Supplementary Materials).

**Figure 5:**
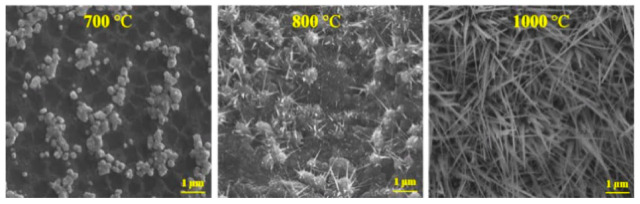
SEM images of Ga/Ag on quartz, which was oxidized at 700, 800, and 1000 °C. At 700 °C, phase segregation was observed, developing multiple nuclei of Ga_2_O_3_ species. At 800 °C, the Ga_2_O_3_ nanoparticles nucleation led to the Ga_2_O_3_ nanowires. At 1000 °C, the growth of the nanowires was controlled by the presence of the dissolved oxygenated Ga species in the molten Ga.

Furthermore, to explore the nucleation and growth of Ga_2_O_3_ nanostructures on quartz from molten gallium in the presence of silver, the oxidation was performed at three different temperatures, i.e., 700 °C, 800 °C, and 1000 °C. Figure 5 shows the various morphologies of Ga_2_O_3_ obtained after oxidation. During the oxidation process, phase segregation occurred, which developed multiple nuclei at the Ga/O_2_ interface during the transport of oxygen to the surface. As the temperature increased, the surface segregation led to the formation of Ga_2_O_3_ nuclei rather than a continuous film. Consequently, single crystalline Ga_2_O_3_ nanowires (Section S2 in Supplementary Materials) were formed and developed from the Ga_2_O_3_ nanoparticles nucleation. The growth of Ga_2_O_3_ occurred from the base of the nanowire using the dissolved oxygenated gallium species in the molten Ga.

During the oxidation of the substrates in the presence of Ag, additional temperature-dependent processes influenced both the nucleation and the growth of nanostructures. Ga_2_O_3_ nucleation in the presence of Ag was greatly influenced by the oxidation temperatures due to the high oxygen diffusivity and solubility in silver at higher temperatures [2,22].

There are other processes, which can occur simultaneously or independent of each other, which could explain the oxygen interaction at the O and Ag interface and the enhancement of the nanowires’ density, shape, and size. First, the silver catalyst could increase the rate of oxygen adsorption as the oxidation temperature increased [27]. Second, at high temperatures, above 626 °C, the annealing of the Ag catalyst could result in high concentrations of adsorbed O_2_ [28]. Initially, O_2_ atoms could have adsorbed in the Ag catalyst to form the surface atomic oxygen and then desorb as O_2_, or they could diffuse by volume diffusion. Third, defects such as vacancies in Ag [29], which were in high concentration due to the sputtering process, could trap oxygen that had been adsorbed on the surface. Oxygen atoms can agglomerate in these defects. There was a direct correlation between the number of defects in Ag and the amount of oxygen it can absorb. Fourth, self-diffusion could particularly impact the distribution of Ag atoms on the substrate surface [30]. Therefore, during diffusion, the Ag atoms could transfer or exchange places easily with no restriction or spread on the surface. Surface diffusion increases as the duration of thermal oxidation increases in a fixed temperature range [31,32]. Fifth, the number of defects could increase at higher temperatures, which directly affects the adsorption of oxygen. Finally, there are the chemical and physical interactions between Ga, Ag, and O, for which comprehensive experimental data do not exist in the literature. In general, the liquid Ga solubility increases with increasing temperature [33]. As a complete system of O–Ag–Ga, after Ag is incorporated into Ga, there is more oxygen in the system available to react with Ga, leading to denser and longer nanowires. At higher temperatures (T ≥ 1000 °C), Ga_2_O_3_ nanowires continue to grow as the diffusivity and solubility of oxygen increases with temperature [2].

### 3.3. Elemental Analysis of Ga_2_O_3_ Nanowires

**Figure 6:**
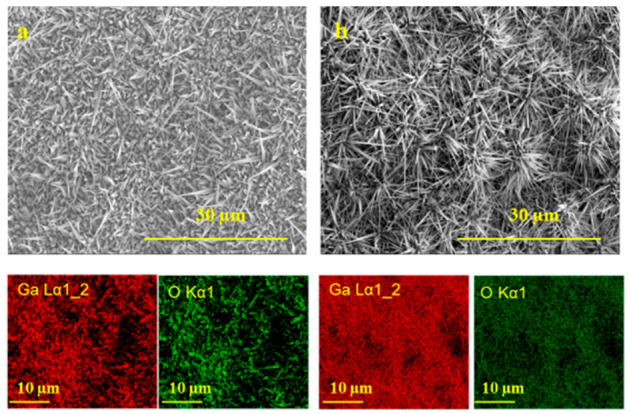
SEM and EDS mapping of grown Ga_2_O_3_ nanostructures on quartz without (**a**) and with (**b**) silver catalyst, which were grown at 1000 °C, along with the associated elemental maps for Ga and O. The density and morphology of the Ga_2_O_3_ nanowires were enhanced in the presence of the Ag catalyst.

**Figure 7:**
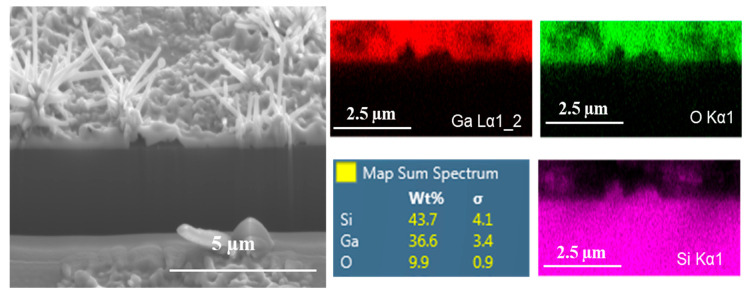
A cross-section of SEM and EDS mapping of grown Ga_2_O_3_ nanostructures on N-Si with the presence of a liquid silver catalyst at 1000 °C and associated elemental maps for Ga, O, and Si. Star-like shape of Ga_2_O_3_ nanowires were observed, with nanowire lengths in the range of 5–10 µm.

**Figure 8:**
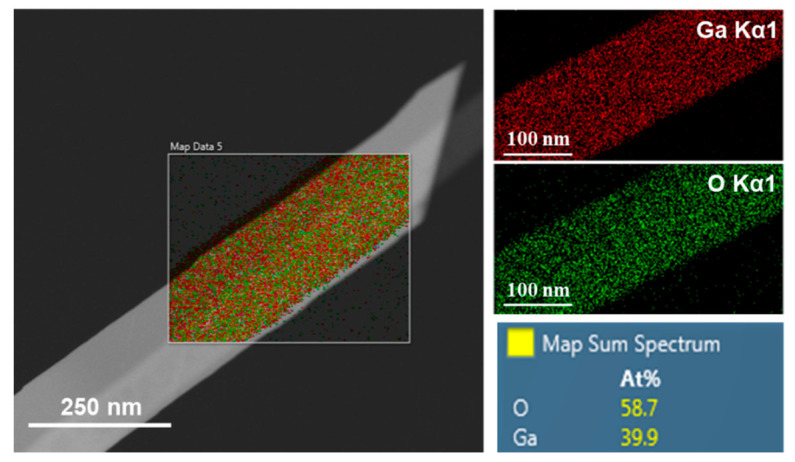
HRTEM image and the corresponding EDS mapping of Ga, O, Ag, and Si of Ga_2_O_3_ nanowire on (100) silicon substrate in the presence of Ag catalyst. A single Ga_2_O_3_ nanowire was observed. Unintentional silicon atoms in Ga_2_O_3_ nanowires could increase the background impurity.

**Figure 9:**
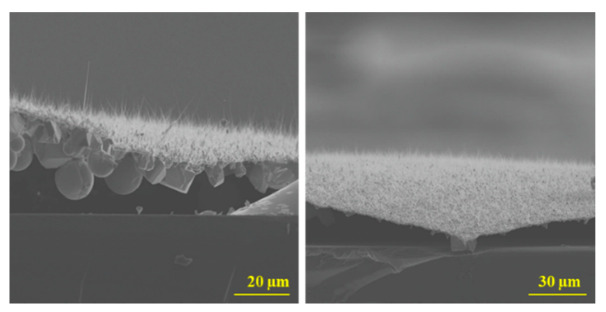
Cross-section SEM images of Ga_2_O_3_ nanostructures on quartz by thermal oxidation of liquid Ga and Ag catalyst at 1000 °C. The cross-section images show the detachment of the Ga_2_O_3_ nanowires layer from the quartz substrate and liquid drops of Ga underneath the film of Ga_2_O_3_. The length of the Ga_2_O_3_ nanowires was in the range of 10–30 µm.

Characterization tools such as energy dispersive spectroscopy, FIB/EDS, and high resolution transmission electron microscopy equipped with an energy-dispersive spectroscopy profile analysis were used to investigate the elemental and chemical microanalysis on quartz and silicon substrate and single Ga_2_O_3_ nanowire (Figure 6, Figure 7, Figure 8 and Figure 9). The SEM images along with the elemental mapping of Ga and O are presented in Figure 6, which present, by comparison, the Ga_2_O_3_ nanostructures on quartz with and without silver obtained by oxidation at 1000 °C for 60 min. Although the EDS detector does not have the capability to detect very low concentrations of Ag, EDS mapping images associated with SEM images showed that the nanowires of Ga_2_O_3_ grown using the Ag catalyst were denser and thinner compared to Ga_2_O_3_ without Ag.

Figure 7 presents the FIB/EDS images obtained on Ga_2_O_3_/Si, which were grown in the presence of silver catalyst at 1000 °C. The focused ion beam equipped with EDS allowed the interface between the oxide and substrate to be viewed. The EDS mapping of the interface cross-section (i.e., the images to the right of the larger SEM image) showed that the Ga_2_O_3_ nanowires of different sizes cover the entire surface of the substrate. Additionally, a fold-like defect was observed in the middle of the SEM image, which was identified as an imperfection of the Si substrate from the associated image of Si mapping.

High resolution transmission electron microscopy (HRTEM) equipped with EDS analysis was used on a Ga_2_O_3_ nanowire grown on Si to further search for silver nanoparticles that were not detected by SEM/EDS but may have been seen by TEM. Figure 8 shows the HRTEM image of a nanowire along with the corresponding elemental mapping for Ga, O, Ag, and Si in the marked area shown in the HRTEM image. Although no Ag nanoparticles were visibly detected on the surface of the nanowire, Ag can be incorporated in Ga_2_O_3_ nanowires. Because the Ag-Si phase diagram shows a eutectic reaction at 845 °C, silicon can therefore react with silver at the oxidation temperature of 950 °C to form a solid solution [22,34]. Consequently, the presence of silicon in Ga_2_O_3_ nanowires may be increased unintentionally, as an impurity that corresponds strongly to n-type conductivity [35]. As a result, the more silver nanoparticles occur, denser and longer nanowires growth could be obtained [22].

### 3.4. Incongruities at the Substrate/Ga_2_O_3_ Interface

Various challenges identified at the substrate/Ga_2_O_3_ interface require further investigation to improve the growth technique needed to produce uniform films of Ga_2_O_3_ nanostructures with controlled morphology. The first issue is related to the different diameter and direction of nanowires and various thickness of the Ga_2_O_3_ film. Second, electrical measurements, such as current-voltage curves, are difficult to perform because some of the gallium may still be in liquid form due to incomplete oxidation. Figure 9 shows droplets of Ga underneath the Ga_2_O_3_ nanowires layer, which was detached from the surface. Detachment of the Ga_2_O_3_ nanowire layer from the substrate has been observed (Figure S2 in Supplementary Materials). This is a major challenge for the process involved in transferring these nanostructures to a different substrate. In addition, other challenges that need to be studied in detail include the study of the effect of surface tension, uneven growth, cracks, delimitation, and the mismatch of the lattice.

Because Ga_2_O_3_ in the form of nanowires is a new material that has only recently been used in devices, the integration of Ga_2_O_3_ NW-based devices with other devices on a chip has still not been developed. For example, researchers are trying to investigate how to control the location and direction of the growth of the Ga_2_O_3_ nanowires. Oxidation is a simple and inexpensive technique, but obtaining nanowire films with controlled morphology requires precise control of the nanowire growth. Additionally, there is a lot of interest in exploring new techniques to grow Ga_2_O_3_ without any surface deformation or surface defects, or to effectively transfer the nanowire film grown on a mother substrate to another substrate in a safe way to minimize the surface damage [36]. If the NW-based device is used as a sensor, this means that the effective sensing surface area is reduced because of the damages due to defects. Therefore, significant and important challenges must be addressed to understand how the Ga_2_O_3_ NWs layer can be reused and transferred on a different substrate in order to fabricate a device with minimum deformations and high-quality coating. One of the interesting challenges is to demonstrate the use of a suitable catalyst for the growth of NWs directly at the location where they will catalyze the growth reaction; however, the catalyst may have some limitations that may affect the performance and compatibility of these devices for electronics and optoelectronics applications.

## 4. Conclusions

Ga_2_O_3_ nanostructure film was grown by thermal oxidation on different substrates in the absence and presence of silver. The morphology of Ga_2_O_3_ nanostructures on different substrates such as p-Si substrate, 250 nm SiO_2_ on n-Si, 250 nm Si_3_N_4_ on p-Si, quartz, and n-Si has been studied. The morphological and elemental analyses of the Ga_2_O_3_ nanostructures showed a high density of long and thin nanowires obtained in the presence of the Ag catalyst. At the oxidation temperature of 1000 °C, the morphology and coverage of the Ga_2_O_3_ NWs showed very good NW features (i.e., high density, long and thin nanowires with sharp tip) which increased the electron transfer. Different electronic and optoelectronic applications can be utilized to study the performance of these ultra-sharp tip nanowires.

## Supplementary Materials

The following are available online at https://www.mdpi.com/2079-4991/10/10/1920/s1, Section S1: Temperature effect on the growth of Ga_2_O_3_ on various substrates. Figure S1: SEM images of the gallium oxide grown on different substrates at 800 and 1000 °C. Gallium oxide nanowires were not observed after the oxidation of gallium bellow 800 °C. Oxidation of gallium at temperatures higher than 1000 °C resulted in a dense growth of nanowires, Section S2: Selected Area Electron Diffraction (SAED), Section S3: Detachment of the Ga_2_O_3_ nanowire layer from the substrate. Figure S2. Cross-section SEM images of Ga_2_O_3_ nanostructures obtained on various substrates by thermal oxidation process at 1000 °C.
