# Supplementary material for: Comparative Study of Growth Morphologies of Ga2O3 Nanowires on Different Substrates
Source: Nanomaterials (Basel). 2020 Sep 25;10(10):1920. doi: 10.3390/nano10101920 (PMC7599797; doi:10.3390/nano10101920)
Supplement: Supplementary file 1 [file nanomaterials-10-01920-s001.pdf]

Supporting Information: Comparative Study of Growth Morphologies of Ga_2O_3 Nanowires on Different Substrates

Badriyah Alhalaili, Ruxandra Vidu, Howard Mao and M. Saif Islam

S1. Temperature effect on the growth of Ga_2O_3 on various substrates.

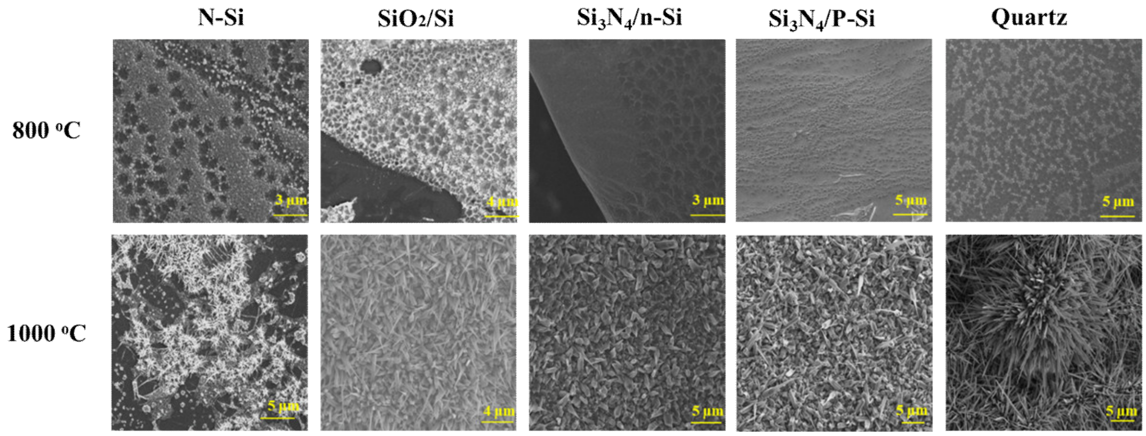

Figure S1. SEM images of the gallium oxide grown on different substrates at 800 and 1000 °C. Gallium oxide nanowires were not observed after the oxidation of gallium bellow 800 °C. Oxidation of gallium at temperatures higher than 1000 °C resulted in a dense growth of nanowires.

S2. Selected Area Electron Diffraction (SAED)

Selected area electron diffraction (SAED) on nanowires grown with and without silver as a catalyst, has been performed to gather further information about the crystal structure of the nanowires. From the XRD results [1] and EDS results in Figures 7,8, it is known that the nanowires are β-Ga_2O_3. In both cases the growth direction is found to be the [−110], with the orthogonal direction as [001]. This finding indicates that the difference in the [020] and [002] peaks shown in Figure 3 [1] was not due to a difference in the orientation of the nanowires, but some other factor instead. Both samples growth with Ag and without Ag showed the same orientation, indicating that the accelerated growth of nanowires in the presence of Ag catalyst does not affect the orientation of the resulting nanowires. In addition to the growth direction, it may be concluded that the nanowires are single crystals in both cases.

S3. Detachment of the Ga_2O_3 nanowire layer from the substrate.

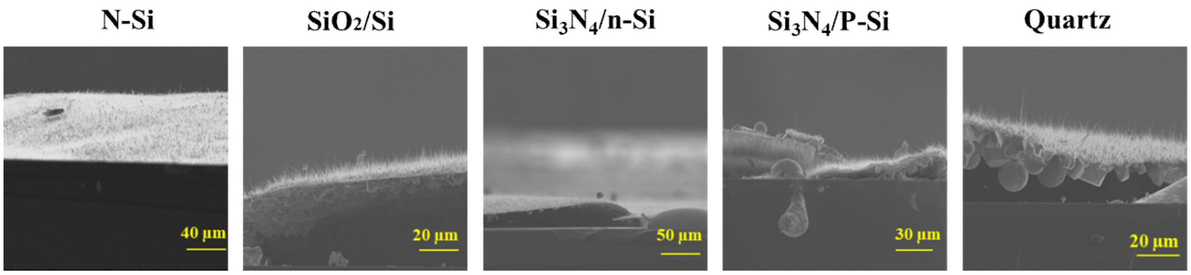

Figure 2. Cross-section SEM images of Ga_2O_3 nanostructures obtained on various substrates by thermal oxidation process at 1000 °C.

References

1. Alhalaili, B.; Bunk, R.; Vidu, R.; Islam, M.S.; Bunk; Vidu Dynamics Contributions to the Growth Mechanism of Ga_2O_3 Thin Film and NWs Enabled by Ag Catalyst. *Nanomaterials* **2019**, *9*, 1272, doi:10.3390/nano9091272.
